# *Cynara scolymus* affects malignant pleural mesothelioma by promoting apoptosis and restraining invasion

**DOI:** 10.18632/oncotarget.4017

**Published:** 2015-06-22

**Authors:** Claudio Pulito, Federica Mori, Andrea Sacconi, Luca Casadei, Maria Ferraiuolo, Maria Cristina Valerio, Raffaela Santoro, Frauke Goeman, Anna Maidecchi, Luisa Mattoli, Cesare Manetti, Silvia Di Agostino, Paola Muti, Giovanni Blandino, Sabrina Strano

**Affiliations:** ^1^ Molecular Chemoprevention Unit, Molecular Medicine Area, Regina Elena National Cancer Institute, Rome, Italy; ^2^ Translational Oncogenomic Unit, Molecular Medicine Area, Regina Elena National Cancer Institute, Rome, Italy; ^3^ Department of Chemistry, University of Rome “La Sapienza”, Rome, Italy; ^4^ Aboca SpA Società Agricola, Sansepolcro, Italy; ^5^ Department of Oncology, Juravinski Cancer Center, McMaster University, Hamilton, Ontario, Canada

**Keywords:** mesothelioma, tumorigenicity, invasion, pathway, apoptosis

## Abstract

Malignant pleural mesothelioma is a poorly treated neoplasia arising from the pleural mesothelial lining. Here we document that the leaf extract of *Cynara scolymus* exerts broad antitumoral effects both *in vitro* and *in vivo* on mesothelioma cell lines. We found that *Cynara scolymus* treatment affects strongly cell growth, migration and tumor engraftment of mesothelioma cell lines. Strikingly, dietary feeding with *Cynara scolymus* leaf extract reduces the growth of mesothelioma xenografted tumors similarly to pemetrexed, a commonly employed drug in the treatment of mesothelioma. In aggregate our findings suggest that leaf extract of *Cynara scolymus* holds therapeutic potential for the treatment of mesothelioma.

## INTRODUCTION

Malignant pleural mesothelioma (MPM) is an aggressive tumour arising from the mesothelial lining of the pleura, which remains compartmentalized for most of the disease course [1] [2]. Current therapeutic approaches include surgery, radiotherapy and chemotherapy. Cisplatin (CDDP) alone or with pemetrexed (PMTX) still represents the current standard of care [3] [4] [5]. Similarly to most solid tumours, the acquired resistance impedes the success of the therapeutic response. Overall patient's survival ranges from 8 and 18 months. Furthermore, there is not any diagnostic tool that allows an early detection of the disease [2] [6]. Given the role played from the asbestos in the etiology of the mesothelioma, this tumor is still considered an occupational disease [7] [8] [9]. Mesothelioma is an orphan disease with an increasing worldwide incidence, especially in the developing countries where the asbestos is still exported and not banned [9] [10]. After inhalation asbestos fibers infiltrate pleural spaces and accumulate in black spots. This leads to the induction of pleural fibrosis that might result in cancer transformation [11] [12]. Within the last five decades, the natural products have continuously contributed to drug discovery and development process [13] [14] [15]. Increasing evidences have shown that natural products, including extracts and isolated chemicals, are multi-targeted and can be considered as a model to approach chronic diseases such as cancer. Actually, most natural agents do not induce high level of toxicity and target simultaneously multiple signalling pathways involved in cell growth, apoptosis, invasion, angiogenesis and metastasis [16] [17]. Since cancer is the result of a deregulation of multiple signalling pathways and natural products elicit multi-targeted activities, the latter could hold a great potential for treating human tumors [18] [6]. The long period between asbestos exposure and the development of disease may be a window of opportunity for chemoprevention or dietary interventions. It has been shown that some natural agents such as coffee, resveratrol, curcumin and butein exert anticancer activities in mesothelioma cellular systems [2] [10] [19] [19] [20]. Observational studies have shown that high adherence to a Mediterranean diet, which encompasses a combination of characteristic foods such as fruits, vegetables fresh produce, fish and seafood, nuts, legumes/pulses and olive oil [21], is associated with a significant reduction in the risk of overall cancer mortality (10%), colorectal cancer (14%), prostate cancer (4%) and aero digestive cancer (56%) [22]. The most diffuse phytochemicals in nature are the classes of polyphenols. For long time, polyphenols have been considered only for their antioxidants properties. Recent data have revealed that polyphenols affect different cell signalling pathways. This also occurs through the modulation of microRNAs thereby affecting the expression of their mRNA targets whose encoded proteins are critical components of different pathways [23] [23]. Globe artichoke (*Cynara scolymus*) is a fundamental component of Mediterranean diet [24]. It has been reported that the edible parts (receptacles with inner and intermediate bracts) and leaves of artichokes represent a potent source of polyphenols fractions. Recent data have revealed that polyphenols affect different cell signalling pathways and exert their anticancer effects along the different steps of carcinogenesis [25] [26] [27]. Furthermore, the main components of *Cynara scolymus* are caffeoylquinic acid derivatives (cynarin and chlorogenic acid), flavonoids (luteolin and apigenin) and bitters (cynaropicrin) [28] [29] [28]. Several *in vitro* and *in vivo* experiments have shown that *Cynara scolymus* exhibits choleretic, hepatoprotective, antibacterial, antinflammatory, antithrombotic and hypocolesterolemic properties [30] [31] [32] [33]. Artichoke extracts are reported to induce apoptosis and cytotoxic effects in cancer cells [34] [35] [36]. In the present report we aimed to demonstrate the anti-cancer activity of the artichoke leaf extract through direct experimental tests, through the normalizing effect of the extract on the cancer metabolic alterations and through the evidence of extract antitumoral activity due to its impact on signalling pathways of oncogenic significance. We found that artichoke leaf extracts (freeze-dried extract prepared as indicated in the methods section by the ABOCA company- http://www.aboca.com) severely affect *in vitro* and *in vivo* mesothelioma tumorigenicity. Indeed, the artichoke leaf extract significantly reduces cell proliferation and colony formation of diverse mesothelioma cell lines. It also promotes apoptosis and restrains mesothelioma cell migration and invasion. It also impairs engraftment and reduces tumor volume of xenografted mesotheliomas. These effects are similar to those induced by pemetrexed. Protein array analyses reveal that the artichoke leaf extract activates distinct set of proteins to those of pemetrexed or cisplatin that might be critical mediators of its antitumoral activities.

## RESULTS

### The artichoke leaf extract inhibits MPM cell growth and proliferation

**Figure 1:**
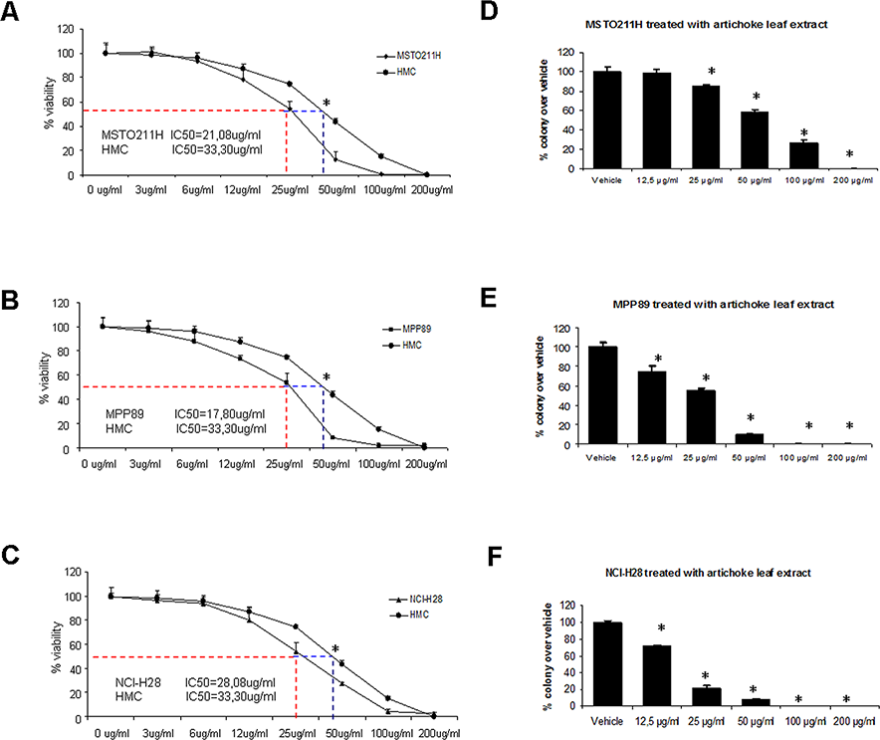
The artichoke leaf extract strongly affects the growth of MPM cells in a dose dependent manner **A, B, C.** Viability of MPM cells (MSTO-211H, MPP89, NCI-H28) and normal untransformed mesothelial cell line (HMC) treated for 72 hrs with *Cynara scolymus* leaf extracts. The IC-50 value was indicated by a dotted line in each panel. Data are represented as mean +/− SD. Statistics (*t*-test): *p* < 0.05. **D, E, F.** Colony forming assay. Histograms showing average colony counts from duplicate experiments. Bars indicate the average of three independent experiments. Statistics (*t*-test): *p* < 0.05.

We aimed first to test the effects of the artichoke extract on mesothelioma cell growth and proliferation. To this end, we treated MSTO-211H, MPP-89 and NCI-H28 mesothelioma cell lines and untransformed mesothelial cells, HMC (Figure 1A–1C) with increasing concentrations (ranging from 3 to 200 μg/ml) of artichoke leaf extract for 72 hrs. We determined the half-maximal concentration of growth inhibition (IC50) for the extract phytocomplex in MPM cells (Figure 1A–1C, Supplementary Table 1). We found that the artichoke extract inhibited cell viability in a dose dependent manner (Figure 1A–1C). Moreover, MSTO-211H cells treated with the artichoke extract showed a change in the cellular morphology both in a short-term assay that in long term as showed in Supplementary Figure 1. In contrast, HMC cells were more resistant to the growth inhibitory effect of the artichoke extract (Figure 1A–1C). Next, we performed a colony-forming assay to evaluate the capability of mesothelioma cells to form colonies after the removal of the extract. We found that the artichoke leaf extract inhibited the colony forming ability of MSTO-211H, MPP-89 and NCI-H28 cells (Figure 1D–1F). Altogether these findings indicated that the artichoke leaf extract treatment is highly effective on cell proliferation and colony forming ability of mesothelioma cell lines.

### The artichoke leaf extract induces apoptosis of MPM cell lines

**Figure 2:**
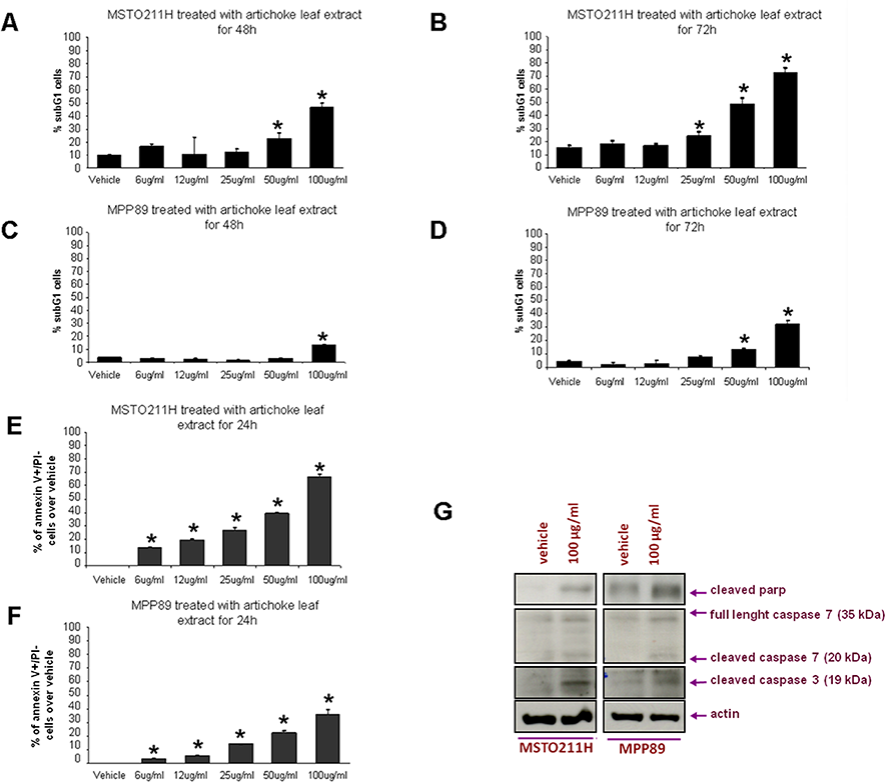
The artichoke leaf extract induces apoptosis of MPM cells **A, B, C, D.** Percentage of subG1 cell population of MPM cells (MSTO-211H and MPP-89) treated with the indicated doses of the artichoke extract for 48 or 72 hrs. Error bars represent mean +/− SD. Statistics (*t*-test): *p* < 0.05. **E, F.** Histograms showing the percentage of Annexin V+/PI− over vehicle. MPM cells (MSTO-211H, MPP-89) were treated at the indicated doses of the artichoke extract for 24. Error bars represent mean +/− SD. Statistics (*t*-test): *p* < 0.05. **G.** Representative protein gel blot of whole cell lysates obtained from MSTO-211H and MPP-89 cell lines treated for 24 hrs with 100 μg/ml of artichoke leaf extract and stained with anti-cleaved parp, anti-caspase-3 and anti-caspase-7 antibodies. Actin staining was used as loading control.

The induction of apoptosis is a pivotal event for successful cancer treatment by natural agents. To this end, we assessed whether the artichoke leaf extract induced apoptosis of mesothelioma cell lines using different approaches. Cytofluorimetric analysis revealed that the artichoke extract induced the appearance of a subG1 peak in MSTO-211H and MPP-89 cells. This effect was dose-dependent (Figure 2A–2D). Furthermore, cells treated with the artichoke leaf extract for 24 hrs became Annexin V-positive in a dose-dependent manner (Figure 2E–2F). We also found that the extract treatment led to increased cleavage of caspase 3, caspase7 and Parp (Figure 2G). Comet assay performed in MSTO-211H and in HMC cells revealed that Cisplatin (7, 5 μg/ml, for 20 h) treatment induced DNA damage of both cell lines (Supplementary Figure 2A, 2B). Interestingly, the exposure to the artichoke leaf extract, used at not apoptotic concentration, 3 μg or 6 μg/ml, did not induce DNA damage (Supplementary Figure 2A, 2B) and reduced that induced by CDDP in HMC cells (Supplementary Figure 2A). Overall, these results clearly demonstrate that the artichoke leaf extract affects cell viability of MPM cell lines by inducing apoptosis.

### The artichoke leaf extract severely impairs migration and invasion of mesothelioma cell lines

It has been previously reported that dietary phenolic acids, monophenols and polyphenols possess inhibitory properties against the invasive and metastatic behaviours of different cancer cells lines.

**Figure 3:**
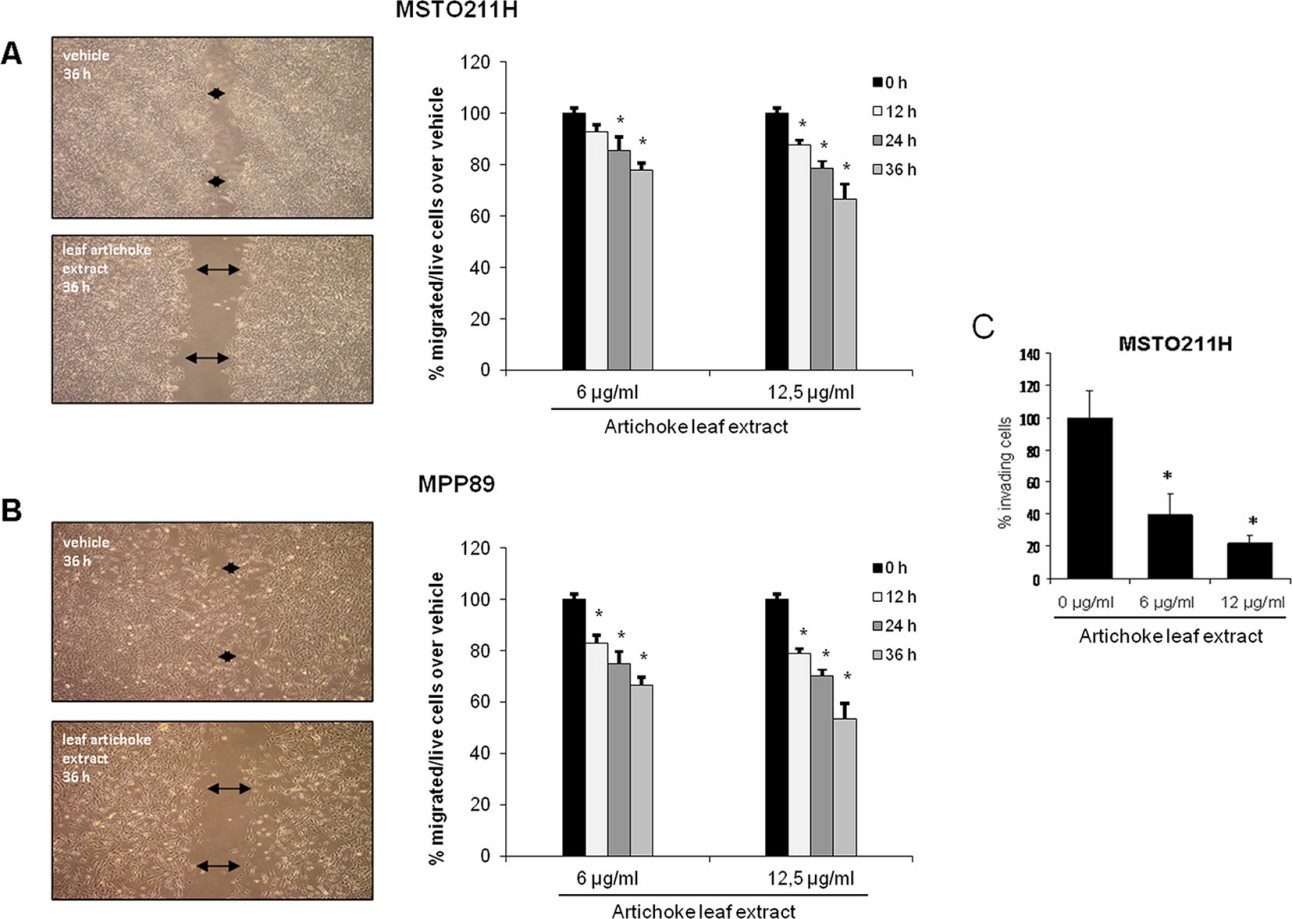
The artichoke leaf extract impairs the migration and invasion of MPM cells **A, B.** Left side: representative micrographs of wound healing closure assays from MSTO-211H and MPP-89 cells treated for 36 hrs with 12, 5 μg/ml of artichoke leaf extract. Right side: histogram showing the healing closure efficiency of the cells treated with vehicle or the artichoke extract (6 μg and 12 μg) after different times of treatment (0, 12, 24 and 36 hrs). Bars indicate the average of three independent experiments. Statistics (*t*-test): *p* < 0.05. **C.** Percentage of invading cells over vehicle. MSTO-211H cells were treated at the indicated doses of extract. Error bars represent mean +/− SD. Statistics (*t*-test): *p* < 0.05.

To this end, we investigated whether the artichoke leaf extract impaired migration and invasiveness of MPM cell lines. First, we performed scratch wound closure assay (Figure 3A, 3B) in MPM cell lines treated with different, not apoptotic, concentrations of the extract. We found that the artichoke extract inhibited, in a time dependent manner, migration of MSTO-211H and MPP89 as wound closure occurred slower than that of the control cells (Figure 3A–3B). As matter of fact, the artichoke leaf extract at the concentration of 6 μg/ml and 12 μg/ml inhibited MSTO-211H cells migration of 50% and 35% respectively as well as in MPP89 cell line (Figure 3A–3B). Second, we performed invasion assay in MSTO-211H cells using a 24-well chamber with a non-coated 8-mm pore size filter in the presence of different concentrations of extract (6 μg and 12 μg/ml). As shown in Figure 3C, the artichoke leaf extract markedly reduced the invasion of MSTO-211H cells. Altogether these results indicated that the artichoke extract inhibited migration and invasion of mesothelioma cell lines.

### The artichoke leaf extract affects *in vivo* tumor growth

**Figure 4:**
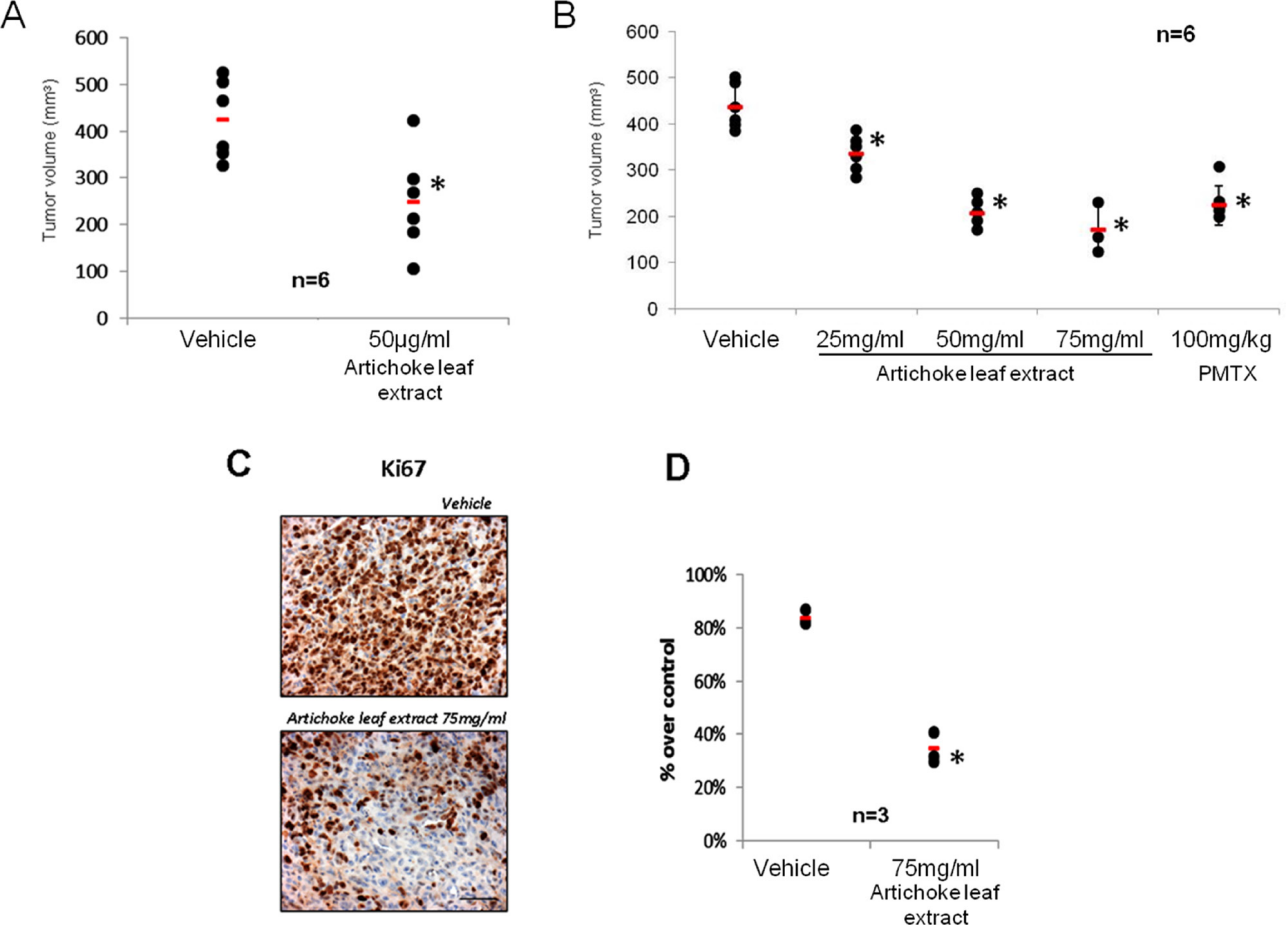
The artichoke leaf extract inhibits *in vivo* mesothelioma tumor growth **A.** Artichoke extract inhibits engraftment of MSTO-211H cells. Suspensions of 2 × 10^6^ MSTO-211H cells were pre-treated with either vehicle or the artichoke leaf extract (50 μg/ml) for 24 hrs and subcutaneously injected into CD1 mice as described in Material and Methods. No further treatment was given to mice. Horizontal bars represent the average tumor volume of the vehicle (*n* = 6) and the artichoke extract (*n* = 6) treated engrafted tumors. Tumors were collected 24 days after MSTO-211H cells injection. Statistics (*t*-test): *p* < 0.05. **B.** The artichoke leaf extract beverage inhibits *in vivo* mesothelioma tumor progression. Tumour volumes of mice (*n* = 6) treated with either vehicle, pemetrexed or the artichoke extract in drinking water are reported. Statistics (*t*-test): *p* < 0.05. **C.** Representative micrographs of the excised tumours stained with anti-Ki-67 antibody. Scale bar, 100 μm. **D.** Histograms showing the percentage of Ki-67 positive nuclei scored in seven fields randomly chosen for each tumours. Statistics (*t*-test): *p* < 0.05.

We aimed to assess *in vivo* the antitumoral effects of the extract. First, we evaluated whether the artichoke leaf extract could affect the engraftment of MSTO-211H cells. To this end, MSTO-211H cells were treated for 24 hrs with vehicle or artichoke extract at 50 μg/ml. Cell suspensions were subcutaneously injected into CD1 mice. As shown in Figure 4A, cells pre-treated with the extract engrafted less efficiently than vehicle-treated cells when inoculated into CD1 mice. We next evaluated the efficacy of the extract to inhibit the growth of xenografted mesotheliomas subcutaneously implanted in CD1 nude mice. To this end, CD1 mice were subcutaneously transplanted with MSTO-211H (2 × 10^6^). At the evidence of tumor progression, mice were randomly divided in five different groups (*n* = 6) and were beverage with vehicle or artichoke leaf extract at different concentrations (25, 50 and 75 mg/ml) in drinking water (Table 2). Dietary feeding of the artichoke leaf extract for 3 weeks reduced significantly and dose dependent the growth of xenografted mesothelioma tumors (Figure 4B). This effect was similar to that induced by PMTX, a drug commonly employed in the treatment of mesothelioma (Figure 4B). To evaluate the proliferation index xenografted tumors were stained for Ki67 expression. Strikingly, we found that xenografted tumors derived from the extract treated mice exhibited significantly lower Ki67 expression than those treated with vehicle (Figure 4C, 4D). We also found that artichoke leaf extracts sensitized MSTO-211H and NCI-H28 mesothelioma cell lines to pemetrexed-induced cell killing (Supplementary Figure 3A-B). Similarly, artichoke leaf extract at 75 mg/ml potentiated the antitumoral effect of pemetrexed on tumor volume of xenografted tumors (Supplementary Figure 3C). Altogether these findings indicate that the artichoke leaf extract can affect *in vivo* the growth of mesothelioma cell lines.

**Table 1:** Artichoke leaf extract components

Classes of Compounds		Levels found in Artichoke freeze-dried extract
**PolyPhenols (%)**	**total** of which lignins total of which tannins total of which Caffeoylquinic acid derivatives total of which Chlorogenic acid of which phenypropanoid derivatives total of which phenols and phenolic acids total of which flavonoids total of which salicilates total	**34.251** 1.98 5.96 10.33 4.07 7.01 0.703 8.27 0.001
**Terpenes (%)**	**total** of which Cynaropicrin	**1.5** 1.5
**Organic Acids (%)**	**total**	**6.3**
**Protein (%)**	**total** of which water soluble proteins	**5.6** 5.6
**Amino Acids fre (%)**	**total**	**0.53**
**Polysaccharides (%)**	**total** of which soluble dietary fiber of which Insoluble dietary fiber	**1.7** 0.8 0.9
**Saccharides (%)**	**total** of which monosaccharides Fructose Glucose of which disaccharides Sucrose of which fructo-oligosaccharides	**24.69** 2.58 8.02 8.89 5.2
**Fats (%)**	**total**	**0.087**
**Phytic acid (%)**	**total**	**0.45**
**Minerals** (*Oligo, micro and macroelements*) (%)	**total**	**9.5**

Different classes of compounds present in the artichoke whole phytocomplex are reported. The phytocomplex is derived from artichokes (*Cynara scolymus*).

**Table 2:** Scheme of treatment

Group	n° of animals	Cell line	n° of cells	via	Volume	Treat. starting	Treat. A	Treat. admin	Treat. schedule	Treat. B	Treat. admin.	Treat. schedule
Group 1	6	MSTO	2*10^6	SC	0.2 ml (Matrigel)		-					
Group 2	6	MSTO	2*10^6	SC	0.2 ml (Matrigel)	After tumor appearance	-			Artichoke (20 ug/ml)	OS	drinkable water
Group 3	6	MSTO	2*10^6	SC	0.2 ml (Matrigel)	After tumor appearance	-			Artichoke (50 ug/ml)	OS	drinkable water
Group 4	6	MSTO	2*10^6	SC	0.2 ml (Matrigel)	After tumor appearance	-			Artichoke (75 ug/ml)	OS	drinkable water
Group 5	6	MSTO	2*10^6	SC	0.2 ml (Matrigel)	After tumor appearance	Pemetrexed (100 mg/kg)	IP	5 consecutive days			
Group 6	6	MSTO	2*10^6	SC	0.2 ml (Matrigel)	After tumor appearance	Pemetrexed (100 mg/kg)	IP	5 consecutive days	Artichoke (20 ug/ml)	OS	drinkable water
Group 7	6	MSTO	2*10^6	SC	0.2 ml (Matrigel)	After tumor appearance	Pemetrexed (100 mg/kg)	IP	5 consecutive days	Artichoke (50 ug/ml)	OS	drinkable water
Group 8	6	MSTO	2*10^6	SC	0.2 ml (Matrigel)	After tumor appearance	Pemetrexed (100 mg/kg)	IP	5 consecutive days	Artichoke (75 ug/ml)	OS	drinkable water

CD1 mice were subcutaneously transplanted with MSTO-211H (2 × 10^6^). At the evidence of tumor progression (when tumor volume reached 60 mm^3^) mice were randomly allocated in placebo and in the artichoke leaf extract-treated groups as described in the table. A control group of mice was intraperitoneally injected with Pemetrexed at the dose of 100 mg/kg for five consecutive days.

### Artichoke leaf extract induces changes in metabolic profiles of MSTO-211H cells

**Figure 5:**
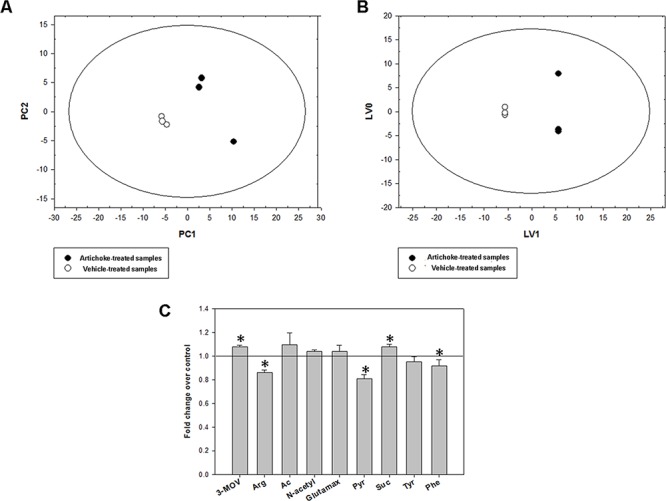
Metabolic response of MSTO-211H cell lines to the artichoke leaf treatment PCA **A.** and O-PLS-DA **B.** models built on the 1H-NMR dataset of media samples from the artichoke extract-treated and vehicle-treated MSTO-211H cell cultures. The score plots show the metabolic differences between the two cell groups. Panel C shows the fold changes relative to vehicle-treated samples (means +/− SD for three independent experiments; (*t*-test): *p* < 0.05) of the most discriminant metabolites between the two groups from the O-PLS-DA model. 3-MOV, 3-Methyl-2-oxovalerate (p); Arg, arginine (c); Ac, acetate; N-Acetyl, group of N-acetyls; Glutamax, glutamax (c); Pyr, pyruvate (c); Suc, succinate; Tyr, tyrosine (c); Phe, phenylalanine (c). “c” and “p” for each metabolite indicate consumption or production, respectively.

Since metabolic alterations are among the hallmarks of a cancer cell we aim to assess whether the artichoke leaf extract could affect the metabolism of MSTO-211H cells. ^1^H-NMR metabolomics profiles of MSTO-211H cell culture media displayed significant differences between artichoke-treated and vehicle-treated cells. Initially, we explored the NMR data through an unsupervised approach such as Principal Component Analysis (PCA) carried out on a dataset of treated and untreated medium samples. The results reported in Figure 5A highlighted significant differences between the two groups on PC1 (*p* = 0.013). Subsequently, to identify the variables (metabolites) that have a major contribution to the discrimination between samples, we analyzed the loadings values from O-PLS-DA with a threshold of 0.8 (Figure 5B, 5C). LV1 included the following variables with the highest correlation levels: 3-methyl-2-oxovalerate, acetate, N-acetyl groups, glutamax and succinate with positive loadings and arginine, pyruvate, tyrosine and phenylalanine with negative loadings. Therefore, considering the net balance of these metabolites, LV1 indicated that artichoke extract induced higher consumption of arginine, pyruvate, tyrosine and phenylalanine as well as a lower consumption of glutamax and succinate. Moreover, artichoke extract-treated cells exhibited higher production of 3-methyl-2-oxovalerate, acetate and N-acetyl groups than vehicle-tread ones. This suggests that exposure to artichoke leaf extract mainly affected the citrate cycle (TCA cycle), arginine and proline metabolism, glutamine and glutamate metabolism, alanine, aspartate tyrosine metabolism and phenylalanine metabolism (Table 3).

**Table 3:** Metabolic pathways perturbed by the artichoke leaf extract in MSTO-211H cells as determined by analysis of OPLS-DA loadings

Metabolite	Artichoke leaf extract vs. vehicle	Kegg Related Pathways*
3-Methyl-2-oxovalerate	higher production	Valine, leucine and isoleucine degradation
Arginine	higher consumption	Arginine and Proline metabolism
		Alanine, aspartate and glutamate metabolism
		Glycine, serine and threonine metabolism
		Citrate cycle (TCA cycle)
Group of N-acetyls	higher production	Arginine and proline metabolism
		Amino sugar and nucleotide sugar metabolism
		Alanine, aspartate and glutamate metabolism
Acetate	higher production	Glycolysis/Gluconeogenesis
		Pyruvate metabolism
		Fatty acid metabolism
Glutamax	lower consumption	D-Glutamine and D-glutamate metabolism
		Purine metabolism
		Pyrimidine metabolism
		Alanine, aspartate and glutamate metabolism
		Arginine and Proline metabolism
Pyruvate	higher consumption	Pyruvate metabolism
		Citrate cycle (TCA cycle)
		Glycolysis
		Pentose phosphate pathway
Succinate	lower consumption	Citrate cycle (TCA cycle)
		Oxidative phosphorylation
		Alanine, aspartate and glutamate metabolism
		Tyrosine metabolism
		Phenylalanine metabolism
		Carbon metabolism
Tyrosine	higher consumption	Tyrosine metabolism
Phenylalanine	higher consumption	Phenylalanine metabolism

* The metabolites are mapped to their respective biochemical pathways as delineated in the Kyoto Encyclopedia of Genes and Genomes (Release 69.0, January 1, 2014; KEGG, http://www.genome.jp/kegg).

### The artichoke leaf extract antitumoral activity impinges on different signalling pathways

**Figure 6:**
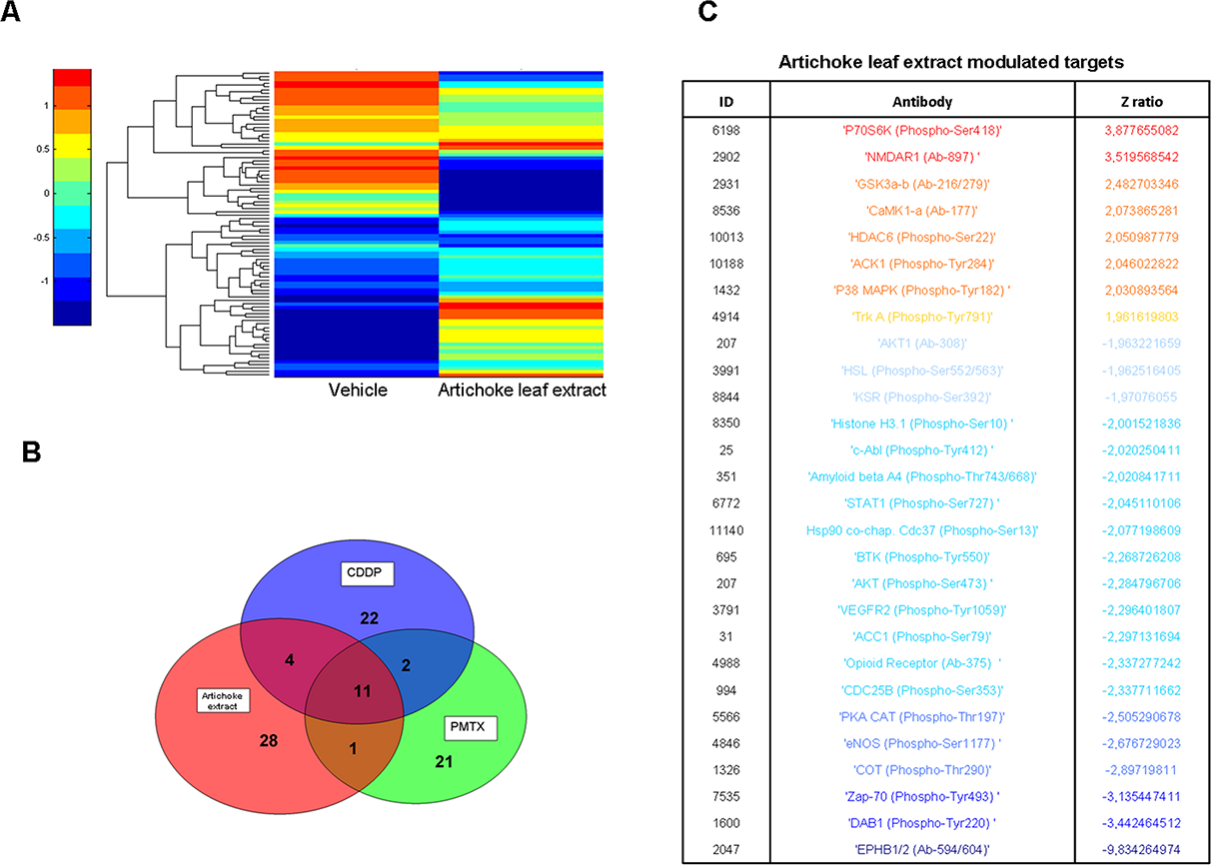
Phosphoprotein analysis of MSTO-211H cells treated with the artichoke leaf extract **A.** Heatmap overall intensities of protein and phospho-protein level of spotted 1318 proteins, after 24 hrs of treatment with vehicle or the artichoke extract (50 μg/ml). Red and blue shadings indicate high and low or undetectable protein levels respectively. **B.** Venn diagram showing the number of proteins and phospho-proteins whose result deregulated after 24 hrs of treatment with the artichoke extract (50 μg/ml), cisplatin (CDDP, 8 μM) or pemetrexed (PMTX, 30 μM). **C.** List of all proteins and phospho-proteins that result deregulated after 24 hrs of artichoke extract treatment. Red and blue colours indicate up and down regulation respectively.

**Figure 7:**
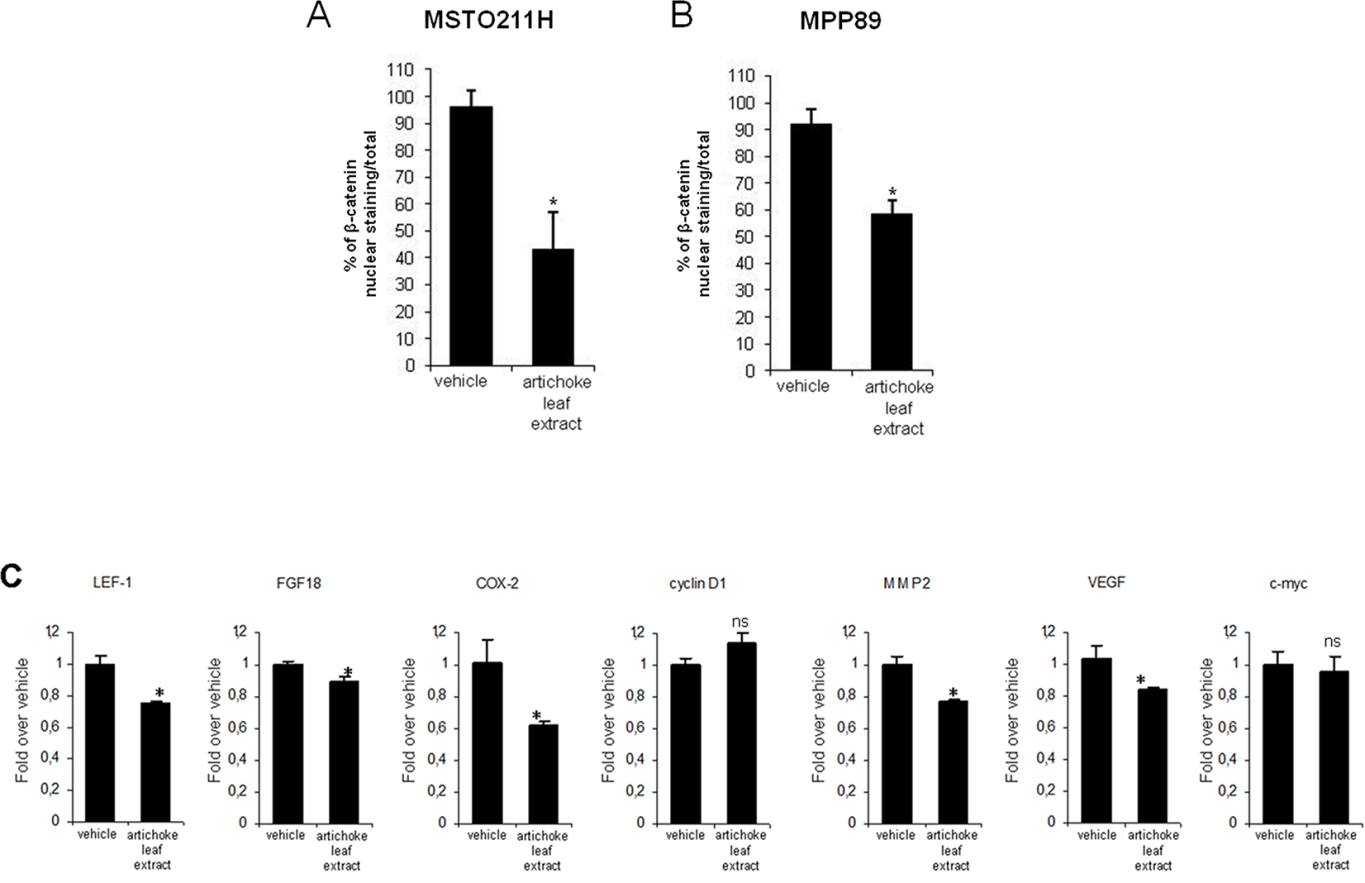
The artichoke leaf extract downregulates Wnt/β-catenin signalling **A-B.** Histograms showing the percentage of β-catenin positive nuclei/total nuclei of MSTO-211H A and MPP-89 cells B treated for 24 hrs with either vehicle or the artichoke extract (50 μg/ml). Bars indicate the average of three independent experiments. Statistics (*t*-test): *p* < 0.05. **C.** Q-PCR for the expression of the indicated Wnt/β-catenin target genes from either vehicle or artichoke leaf extract-treated MSTO 211H cells (50 μg/ml, 24 hrs). Bars indicate the average of three independent experiments. Statistics (*t*-test): *p* < 0.05.

Since the artichoke extract appears to exert broad anti-tumoral effects on mesothelioma cell lines, we aimed to assess which signalling pathways might be involved. To this end, we performed a phospho-antibody array containing 1318 antibodies representative of key proteins involved in over than 30 signalling pathways altered in cancer among which proliferation, apoptosis, invasion, migration, metabolism and angiogenesis. MSTO-211H cells were treated for 24 hrs with the artichoke extract (50 μg/ml) or vehicle and the derived protein lysates were used to probe the antibody array. The heatmap shown in Figure 6A represented the signal intensities of those proteins whose levels of expression or phosphorylation were changed upon the artichoke leaf extract treatment. Among them, we found a group of proteins, which included p70S6k (phospho-Ser 418), NMDAR1 (Ab-897), CAMK1-a (Ab-177), HDAC6 (phospho-Ser22), ACK1 (phosphoTyr284), p38 MAPK (phosphor Tyr-182) that were upregulated and phosphorylated upon the extract treatment compared to vehicle (Figure 6C). Strikingly, the artichoke leaf extract treatment activated key proteins such as p53, BAX p38, and Caspase 3 leading to apoptosis and downregulated survival and pro-tumorigenic factor among which c-Abl, STAT1, AKT, and VEGFR2 (Figure 6C) [37] [38]. By comparing the pattern of modulated proteins in MSTO-211H cells treated with Pemetrexed or Cisplatin we found that the artichoke extract modulated distinct and common sets of proteins when compared to the two drugs respectively (Figure 6B and Table 4). Among the proteins upregulated by the artichoke treatment, GSK 3a-β (Ab-216/279) appear to be pivotal components of multiple signalling pathways such PI3K/PTEN/AKT/mTORC1, Ras/Raf/MEK/ERK, Hedgehog, Notch and WNT. Since aberrant activation of the WNT signalling pathway plays a role in mesothelioma tumorigenesis we investigated whether the treatment with the artichoke extract of MPM cell lines modulated the expression of diverse components of this pathway. We found that the artichoke leaf extract reduced β-catenin nuclear staining in two mesothelioma cell lines (Figure 7A–7B). This paired with the reduced expression of well-known β-catenin target genes such as LEF1, FGF8, COX2, MMP2 and VEGF transcripts (Figure 7C) [39]. Altogether these findings highlight the possibility that the broad antitumoral activities of the artichoke leaf extract on mesothelioma cell lines might occur through the modulation of diverse signalling pathways commonly altered in human cancers.

**Table 4:** List of deregulated or phosphorylated proteins upon CDDP, PMTX or artichoke leaf extract treatment

Pemetrexed		Artichoke leaf extract		Cisplatin	
UP	DOWN	UP	DOWN	UP	DOWN
-PKC theta (Ab-538) -NFkB-p105/p50 (Phospho-Ser337) -LYN (Ab-507) -MAPKAPK2 (Ab-334) -FGFR1 (Ab-654) -Abl1 (Ab-204) -DAXX (Ab-668) -IKK-a/b (Ab-176/177) -RAD52 (Ab-104) -Merlin (Ab-10) -Rb (Phospho-Ser780) -MAPKAPK2 (Phospho-Thr334) -Ephrin B1/B2/B3 (Phospho-Tyr324) -HCK (Ab-410) -DAXX (Phospho-Ser668) -AKT1 (Ab-129) -CD32 (FcgammaRIIb) (Ab-292) -NFkB-p65 (Phospho-Ser468) -NFkB-p100 (Phospho-Ser872) -RapGEF1 (Phospho-Tyr504) -FLT3 (Phospho-Tyr969) -BAX (Ab-167) -HDAC1 (Ab-421) -Caspase-3 (Ab-150) -Mst1/Mst2 (Ab-183) -p53 (Phospho-Ser9) -SREBP-1 (Phospho-Ser439) -PFKFB2 (Phospho-Ser483) -CASP1 (Ab-376) -GRB10 (Ab-67) -Tyrosine Hydroxylase (Phospho-Ser31) -PAK2 (Ab-192)	-Ezrin (Phospho-Tyr478) -EPB41 (Ab-418/660) -Dok-1 (Phospho-Tyr362)	-HDAC6 (Phospho-Ser22) -ACK1 (Phospho-Tyr284) -P38 MAPK (Phospho-Tyr182) -P70S6K (Phospho-Ser418) -GSK3a-b (Ab-216/279) -CaMK1-a (Ab-177) -NMDAR1 (Ab-897) -Trk A (Phospho-Tyr791) -eNOS (Phospho-Ser1177) -BAX (Ab-167) -P38 MAPK (Ab-322) -Caspase-3 (Ab-150) -CD19 (Ab-531) -Mst1/Mst2 (Ab-183) -p53 (Phospho-Ser9) -SREBP-1 (Phospho-Ser439) -MAP3K7/TAK1 (Ab-439) -PFKFB2 (Phospho-Ser483) -CASP1 (Ab-376) -GRB10 (Ab-67) -'Tyrosine Hydroxylase (Phospho-Ser31) -IkB-alpha (Phospho-Ser32/36) -PAK2 (Ab-192)	-Histone H3.1 (Phospho-Ser10) -HSL (Phospho-Ser552/563) -Hsp90 co-chaperone Cdc37 (Phospho-Ser13) -AKT (Phospho-Ser473) -PKA CAT (Phospho-Thr197) -DAB1 (Phospho-Tyr220) -VEGFR2 (Phospho-Tyr1059) -AKT1 (Ab-308) -EPHB1/2 (Ab-594/604) -Opioid Receptor (Ab-375) -Amyloid beta A4 (Phospho-Thr743/668) -c-Abl (Phospho-Tyr412) -COT (Phospho-Thr290) -KSR (Phospho-Ser392) -Zap-70 (Phospho-Tyr493) -STAT1 (Phospho-Ser727) -ACC1 (Phospho-Ser79) -CDC25B (Phospho-Ser353) -BTK (Phospho-Tyr550) -NFkB-p65 (Phospho-Ser468) -Dok-1 (Phospho-Tyr362) -ATP-Citrate Lyase (Phospho-Ser454)	-VEGFR2 (Phospho-Tyr1054) -HER2 (Ab-877) -P38 MAPK (Ab-180) -Ras-GRF1 (Phospho-Ser916) -Smad2 (Ab-245) -RapGEF1 (Phospho-Tyr504) -FLT3 (Phospho-Tyr969) -BAX (Ab-167) -P38 MAPK (Ab-322) -Caspase-3 (Ab-150) -CD19 (Ab-531) -Mst1/Mst2 (Ab-183) -p53 (Phospho-Ser9) -SREBP-1 (Phospho-Ser439) -MAP3K7/TAK1 (Ab-439) -PFKFB2 (Phospho-Ser483) -CASP1 (Ab-376) -GRB10 (Ab-67) -Tyrosine Hydroxylase (Phospho-Ser31) -IkB-alpha (Phospho-Ser32/36) -PAK2 (Ab-192)	-Smad1 (Ab-465) -Cytokeratin 18 (Ab-52) -HER2 (Phospho-Tyr877) -EGFR (Phospho-Tyr1092) -G3BP-1 (Phospho-Ser232) -MEK1 (Phospho-Ser298) -Smad3 (Ab-213) -Tau (Phospho-Ser356) -Tau (Phospho-Thr205) -Androgen Receptor (Phospho-Ser213) -NMDAR1 (Phospho-Ser897) -PLCG1 (Phospho-Tyr1253) -Rb (Phospho-Ser807) -Synaptotagmin (Phospho-Ser309) -p21Cip1 (Ab-145) -Smad1 (Phospho-Ser465) -VAV2 (Ab-142) -Dok-1 (Phospho-Tyr362) -ATP-Citrate Lyase (Phospho-Ser454)

The list of proteins whose result induced or downregulated by Cisplatin, Pemetrexed or the artichoke extract treatment compared to the vehicle is shown. In italics are reported the proteins which were modulated in more than one treatment.

## DISCUSSION

Malignant pleural mesothelioma is an extremely rare, highly lethal tumor: mesothelioma is highly resistant to current chemotherapeutic agents, thus the disease-free-survival of the treated patients is very poor. Here we investigated whether, a phytocomplex derived from Artichoke leaves extracts, affects malignancy of pleural mesothelioma. It has been previously reported that artichoke phenol extracts can promote apoptosis and impair invasiveness of different cancer cell lines [34–36]. We found that the artichoke extract exerts *in vitro* and *in vivo* a broad anti-tumoral effect by strongly affecting MPM growth, migration and invasion. Next-generation sequence technologies have tremendously contributed to decipher tumor landscape and to dissect tumor heterogeneity. This has further established that cancer is the consequence of a broad array of genetic and epigenetic alterations. Modern drugs in the form of single-chemical entities have been successful in the treatment of acute conditions such as infectious diseases. Drug action is fast and predictable, leading to a favourable resolution of a critical situation in a short period of time. The situation is markedly different when treating complex chronic conditions such as cancer, type 2 diabetes and cardiovascular diseases. In particular considering cancer development, carcinogenesis process is multistep (that is, it results from accumulated genetic and epigenetic alterations), multipath (that is, multiple functional pathways are involved, such as self-sufficiency in growth signals, insensitivity to anti-growth signals, apoptosis evasion, limitless replicative potential, tissue invasion and metastasis and sustained angiogenesis), and multifocal (both multiclonal - e.g., field cancerization - and clonal expansion leading to intraepithelial spread). More than 250 population-based studies, including case–control and cohort studies, indicate that people who eat about five servings of fruit and vege day have approximately half the risk of developing cancer, particularly cancers of the digestive and respiratory tracts, of those who eat fewer than two servings. The NCI has identified about 35 plant-based foods that possess cancer-preventive properties. These include garlic, soybeans, ginger, onion, turmeric, tomatoes and cruciferous vegetables (for example, broccoli, cabbage, cauliflower and Brussels sprouts). Thus, the plants contain numerous substances, some of them pharmacologically active other inert but still potentially interacting with the active ones to potentiate the effectiveness as anticancer agents. The effectiveness as anticancer agent of a given plant could be endowed in its complexity (number of composing substances) coupled with multi-targeting activity. In aggregate this might allow concomitant untangling of diverse cancer pathways. Congruently, both metabolomics and antibody array analysis of MPM cells revealed that the artichoke leaf extract affects concomitantly different signalling pathways. We found that the extract affects β-catenin nuclear expression which pairs with the downregulation of the expression of its target genes such as LEF-1, FGF18, COX-2, MMP2 and VEGF. It was previously shown that direct targeting of β-catenin sensitizes mesothelioma cell lines to the treatment with cytotoxic drugs [40]. WNT is together with RAS, PI3K, Hippo and BCL among those pathways that are mostly altered in mesotheliomas [41]. We also found that the artichoke extract induces phosphorylation of the tumor suppressor protein p53 on Ser9. Saito *et al*., have shown that phosphorylation of p53 on Ser 46, as well as on Ser 9 was dependent from ATM protein kinases in response to ionizing radiation [42]. This led to p53 stabilization, transcriptional activation and induction of apoptosis. ATM-mediated phosphorylation on Ser 15 and at nearby residues including Ser 6 and Ser 9 enhanced p53 apoptotic activities. Piccolo's group has shown that p53 phosphorylation on Ser 9 was instrumental for the activation of the TGF-β cytostatic program [43]. AKT/PKB is frequently activated in tumors and is an important player for signalling pathways that regulate growth and survival. Fully active AKT/PKB mediates numerous cellular functions including metabolism, angiogenesis, growth, proliferation, survival, protein synthesis, transcription, and apoptosis. We found that artichoke leaf extract reduced both protein levels of AKT1 and of its phosphorylation at Ser473 that enables fully activation of the signalling axis involving insulin receptor, IRS and PI3 kinase. This pairs with our metabolomics data indicating that artichoke leaf extract affects the citrate cycle (TCA cycle), arginine and proline metabolism, glutamine and glutamate metabolism, alanine, aspartate and glutamate metabolism, tyrosine metabolism and phenylalanine metabolism (Table 3). Thus, artichoke leaf extract might impact on crucial metabolic pathways that are aberrantly activated in human cancers. Altogether these evidences contribute to categorize artichoke leaf extract as a natural product with broad antitumoral activity on malignant pleural mesothelioma. Due to the evidence that the signalling pathways altered in mesothelioma and affected by artichoke leaf extracts are common to other human malignancies we might expect that cynara scolymus could exert antitumoral effects on other types of human cancers (Supplementary Figure 5). Data shown in Figure 1A-F and Supplementary Figures 2A-B and 3A-C provide *in vitro* and *in vivo* evidence for a co-treatment of artichoke leaf extracts with either cisplatin or pemetrexed. Indeed, artichoke leaf extract, unlike cisplatin that provoked DNA damage to both mesothelial (HMC) and mesothelioma cell lines, exerted a more pronounced apoptotic effects on MPM cell lines than HMC. Interestingly artichoke leaf extracts reduced DNA damage of HMC cells upon cisplatin treatment, thereby suggesting that its co-treatment with either cisplatin or pemetrexed not only potentiates their antitumoral effects but might also confers selectivity toward tumor cells. Natural products are also the leading compounds for chemopreventive strategies. This is mainly based on the evidence that are well tolerated and have no or very minimal side effects on the subjects enrolled in chemoprevention trials. MPM is an occupational disease with a latency up to 40–50 years that represents an extraordinary time window for a chemopreventive approach. Indeed, a Phase II clinical trial (NCT02076672) aiming to investigate the anti-cancer activity of artichoke leaf extract in an asbestos-exposed population is currently enrolling participants in Ontario.

## MATERIALS AND METHODS

### Cell lines

The human MPM cell lines MSTO-211H, NCI-H28, MPP89 were purchased from the ATCC (Rockville, MD). HMC, human mesothelial cells, were purchased from Tebu-Bio (Le Perray en- Yvelines, France). All MPM cell lines, were cultured as monolayers at 37°C and 5% CO2 in DMEM/F12 + GLUTAMAX (InVitrogen, Carlsbad, CA) supplemented with 10% non-heat inactivated FBS (Gibco, Life Technologies, USA) and 0, 5 Unit/ml insulin (Humulin, Eli Lilly and Company, Indiana, USA), while HMC were cultured in Mesothelial cell basal medium (Zen-Bio, Research Triangle Park, NC).

### Extraction procedure and phytochemical characterization

Frozen artichoke leaf samples were extracted (DER from 5 to 6:1) by hydro-alcoholic procedure (ETOH 50%). The phytocomplex consists of a partially purified mixture of polyphenolic compounds and terpenes extracted from artichoke (Cynara scolymus L.) enriched in caffeoylquinic acids, chlorogenic acid and cynaropicrine. It is a pending Aboca's patent (RM2014A000685). It was submitted to a complete characterization of the composition by means of metabolomic analysis (LC-ESI/MS) (as previously described [28] and by quantitative analysis of caffeoylquinic acids (SFM), chlorogenic acid (HPLC-UV) and cynaropicrine (HPLC-UV). The complete chemical classes of compounds (phenols, terpenes, fats, proteins, amino acids, minerals, polysaccharides, etc.) addictionally analysed and presents in the phytocomplex are listed in Table 1.

### Chemicals and antibodies

Artichoke capsules (Aboca, Sansepolcro, Italy); Pemetrexed (ALIMTA, Eli Lilly and Company, Indiana, USA) and Cisplatin (Pfizer Pharmaceuticals Group, New York, USA) were dissolved according to the manufacturer's instructions. The following primary antibodies were used: anti-cleaved PARP (Asp214) (Cell Signaling, # 9541); anti-caspase 7 (Cell Signaling, #9492); anti-caspase 3 (Enzo life Science, #31A1067); anti-beta actin (A-2228, SIGMA); β-catenin antibody (clone CAT-5H10, # 18–0226. Zymed). Secondary horseradish peroxidase-conjugated was purchased from Santa Cruz; secondary antibody for immunofluorescence Alexa Fluor 594 (mouse) conjugated was obtained from Molecular Probes (Inc, Eugene, OR, USA). ECL reagent (Amersham, GE Healthcare, Piscataway, NJ, USA) was employed for the chemo-luminescence detection. Annexin V FITC (0, 2 μg/ml) (Abcam, ab-63556); DAPI staining (Sigma) was used for nuclear detection.

### RNA processing and qRT-PCR

Total RNA from mesothelioma cell lines was extracted by using Trizol Reagent following manufacturer's instructions (InVitrogen).

The first-strand cDNA was synthesized according to the manufacturer's instructions (M-MLV RT kit, Invitrogen). Gene expression was measured by real-time PCR using the FastStart SYBR Green Master Mix (Applied Biosytems) on a 7900HT instrument (Applied Biosystems). Sequences of qPCR primers are LEF-1 Fw: 5′-AGCGAATGTCGTTGCTGAGTGTA-3′, Rv 5′-CT CTTGCAGACCAGCCTGGATAA-3′. FGF18 Fw: 5′-CT CTACAGCCGGACCAGTG-3′, Rv: 5′-CCGAAGGTGTC TGTCTCCAC-3′. ACTIN Fw: 5′-GGCATGGGTCAGA AGGATT-3′, Rv: 5′-CACACGCAGCTCATT GTAGA AG-3′. C-MYC Fw: 5′-CTCCTGGCAAAAGG TCAGA G-3′, Rv: 5-TCGGTTGTTGCTGATCTGTC-3′. COX2 Fw: 5′-GAATGTTCCACCCGCAGTACA-3′, Rv: 5′-GCATAAAGCGTTTGCGGTAC-3′. VEGF Fw 5′-CGA GGGCCTGGAGTGTGT-3′, Rv: 5′-CGCATAATCTGCAT GGTGATG-3′. CyclinD1 Fw: 5′-GCCCTCGGTGTCC TACTTC-3′, Rv: 5′-AGGAAGCGGTCCAGGTAGTT-3′. MMP2 Fw: 5′-AAGTCTGGAGCGATGTGACC-3′, Rw: 5′-GAGTCCGTCCTTACCGTCAA-3.′

### Cell viability assay

Cell viability of treated cells was assessed using ATPlite assay (Perkin Elmer, Massachusset, USA) accordingly to the manufacturer's instructions. Cell viability tests were carried out to determine the efficacy of *Cynara scolymus* concentrations using different plant fractions and lot numbers. Cells (8 × 10^2^ cells) were seeded in 96 well-plates and cultured for 24 hrs and treated for 72 hrs with *Cynara scolymus* leaf extracts. Each plate was evaluated immediately on a microplate reader (Expire Technology, Perkin Elmer).

### Clonogenic assays

MPM cell lines were grown at 70% confluence and treated with *Cynara scolymus* leaf extracts or with vehicle. Sixteen hrs later, cells were detached and seeded at 600 cells per 6 well into six-well dishes (Corning-Costar, Tewksbury, MA, USA) in drug-free media. Fresh media (25%) was added every three days. After 15–21 days, colonies were stained with crystal violet and colonies counted.

### Apoptosis detection

To determine the effect of artichoke leaf extracts on the cell cycle FACS analysis was carried out. For propidium iodide (PI) staining, cells were seeded in 6-well plates at a density of 10^4^ cells/ml. After 24 hrs cells were treated with indicated plant extract concentrations for different time intervals. Floating and attached cells were harvested, washed in PBS, fixed in ice-cold ethanol (70% v/v) and stored at −20°C. For the analysis, cells were washed in PBS and incubated with RNase A (1 mg/ml) and PI (40 μg/ml) was added. For PI/Annexin V double staining treated cells were harvested and suspended in binding buffer (HEPES pH 7.4, CaCl2 2.5 mM, NaCl 140 mM). Aliquots of cells were incubated for 15 min with Annexin V FITC (0, 2 μg/ml) (Abcam, ab-63556) and PI (5 mg/ml) (Invitrogen). For each FACS analysis, 3 × 10^3^ events for each sample were analyzed. Flow cytometry analyses were carried out with Easycyte 8HT (Guava, Millipore) followed by analysis using InCyte software (Millipore).

### Transwell invasion assay

Migration assay was performed using a 24-well Boyden chamber with a non-coated 8-mm pore size filter in the insert chamber (BD Falcon, Franklin Lakes, NJ, USA). Cells were suspended in 0.5 ml DMEM/F12 media without containing FBS and seeded into the insert chamber. Cells were allowed to migrate for 24 hrs into the bottom chamber containing 0.5 ml of DMEM/F12 media containing 10% FBS in a humidified incubator at 37°C in 5% CO2. Migrated cells that attached to the outside of the filter were visualized by staining with DAPI and counted. The average number of cells per field was expressed as percentage of the control after normalizing for cell number.

### Wound–healing migration assay

MPM cells were grown to 80% of confluence in 6-well tissue culture plates and wounded with a sterile 10-mL pipet tip to remove cells. PBS washing was used to remove loosely attached cells. The progression of migration was at photographed at different times under a light microscope. The number of cells migrated into the scratched area was calculated.

### Comet assays

Cells were pre-treated with or without 3 μg or 6 μg/ul of *Cynara scolymus* for 16 hrs. After that, cells were treated or not with CDDP, 7.5 μg/ml, for 20 hrs. After treatment, cells were detached with trypsin and embedded in 1% low melting agarose (Sigma) in phosphate-buffered saline and spread onto microscopy slides coated previously with 1% agarose (Bio-Rad). Cells were lysed in the lysis solution (2.5 M NaCl, 100mM ethylenediaminetetraacetic acid, 10mM Tris base, 8 g/l NaOH, 1% Triton X-100, 10% dimethyl sulfoxide) for 1 h at room temperature and then run in running solution (300mM NaOH, 1mM ethylenediaminetetraacetic acid, pH 13.0) for 30 min at 25V and 250 mA. DNA was equilibrated with 0.4M Tris (pH 8.0) and slides were dried with methanol. DNA was stained with propidium iodide (Sigma) and pictures were taken using 63 × magnification at an Axiovert 200 M microscope and Axiovision acquisition program (Zeiss). At least 300 cells were scored for each slide.

### Western blot analysis

Cell lysis was performed on ice for 30 min in NP40 lysis buffer (50 mM Tris-HCl pH 7.4, 150 mM NaCl, 1% NP-40, 1 mM EGTA, 1 mM EDTA) supplemented with protease and phosphatase inhibitors (5 mM PMSF, 3 mM NaF, 1 mM DTT, 1 mM NaVO4). Equal amounts of total proteins extracts (30 μg) were resolved by 8% denaturing SDS polyacrylamide gel electrophoresis (SDS-PAGE), and transferred for 2 hrs to polyvinylidene difluoride membrane. Membranes were blocked in 5% milk-TBS-0.05% Tween 20 for 1 hour and incubated overnight with the specific primary antibodies (see chemicals and antibodies section).

### Immunofluorescence microscopy

Briefly, MSTO-211H cells were seeded into eight-chamber culture slides (BD Falcon). The next day, cells were rinsed with ice-cold PBS buffer and fixed with 4% paraformaldehyde for 10′ at room temperature and then permeabilized with 1% Triton X-100. The cells were incubated overnight with the indicated antibody. The day after, cells were washed with cold PBS three times for 3 min each and stained for 2 hrs with a secondary antibody Alexa 488-conjugated goat anti-mouse IgG (Molecular Probes Cells) and counterstained with DAPI (40, 6-diamidino-2-phenylindole, dihydrochloride). Cells were examined under a Zeiss LSM 510 laser scanning fluorescence confocal microscope (Zeiss, Wetzlar, Germany).

### Sample preparation for NMR spectroscopy

Each medium sample (2 ml) was lyophilized, then dissolved in 700 μl of 1 mM TSP [sodium salt of 3-(trimethylsilyl) propionic-2, 2, 3, 3-d_4_ acid], 10 mM sodium azide D_2_O phosphate buffer solution (pH = 7.4) and finally homogenized by vortex mixing for 1 min. After centrifugation (10 min, 10.000 RCF at 22°C), 600 μl of each resulting supernatant was transferred to a 5-mm NMR tube and used for the NMR analysis.

### ^1^H-NMR spectroscopy

2D ^1^H *J*-resolved (JRES) NMR spectra were acquired on a 500 MHz Varian/Agilent spectrometer (Agilent, Santa Clara, CA) using a double spin echo sequence with 4 transients per increment for a total of 32 increments. These were collected into 16 k data points using spectral widths of 6 kHz in F2 and 40 Hz in F1. There was a 2.0 s relaxation delay. Each FID was Fourier transformed after a multiplication with sine-bell/exponential function in the F2 dimension and a sine-bell function in the F1 dimension. JRES spectra were tilted by 45°, symmetrised about F1, referenced to TSP at d_H_ = 0.0 ppm and the proton-decoupled skyline projections (p-JRES) exported using Agilent VNMRJ 3.2 software. Metabolites responsible for the separation between treated and untreated samples were identified using an in-house NMR database and Chenomx NMR suite v. 7.7 (Chenomx Inc., Alberta, Canada).

### NMR spectra pre-processing treatment

The 1D skyline projections exported were aligned and then reduced into spectral bins with ranging from 0.01 to 0.02 ppm by using the ACD intelligent bucketing method (1D NMR Manager software (ACD/Labs, Toronto, Canada). To compare the spectra, the integrals derived from the binning procedure were normalized to the total integral region, following exclusion of bins representing the residual water peak (4.33–5.17 ppm) and the TSP peak (0.5–0.5 ppm).

The resulting data was used as input for multivariate analysis: Principal Component Analysis (PCA and Orthogonal projections to latent structures discriminant analysis (OPLS-DA) were performed using SIMCA-P + version 12 (Umetrics, Umea, Sweden).

### Phospho-protein profiling by the phospho explorer antibody microarray

The Phospho Explorer antibody microarray, which was designed and manufactured by Full Moon Biosystems, Inc. (Sunnyvale, CA), contains 1318 antibodies [44]. Each of the antibodies has two replicates that are printed on a coated glass microscope slide, along with multiple positive and negative controls (Supplementary Figure 4). The antibody array experiment was performed using Full Moon Biosystems, according to their established protocol. In brief, cell lysates obtained from MSTO-211H treated with the artichoke extract at 50 μg/ml or with Cisplatin at 8 μM or Pemetrexed at 30 μM of concentrations for 24 hrs, were biotinylated with the antibody array assay kit (Full Moon Biosystems, Inc.). The antibody microarray slides were first blocked with a blocking solution (Full Moon Biosystems, Inc.) for 30 min at room temperature, rinsed with Milli-Q grade water for 3–5 min. The slides were then incubated with the biotin-labeled cell lysates in coupling solution (Full Moon Biosystems, Inc.) at room temperature for 2 hrs. The array slides were washed 4 to 5 times with 1x Wash Solution (Full Moon Biosystems, Inc.) and rinsed extensively with Milli-Q grade water before detection of bound biotinylated proteins using Cy3-conjugated streptavidin. Each slide (containing six replicates) hybridized and Cy3 fluorescence acquired by microarray scanner with a scan resolution of 10 mm (Agilent Technologies). The images were quantified using Agilent Feature Extraction (AFE) software (Agilent Technologies). The fluorescence signal of each antibody was obtained from the fluorescence intensity of this antibody spot after subtraction of the blank signal (spot in the absence of antibody).

### Statistical analysis

Bionformatic analysis was performed with Matlab (The MathWorks Inc.). Z score transformation was used to express the background corrected spot intensity values as unit of a standard deviation from the normalized mean of zero [45]. Features were selected basing on Z ratios calculated by taking the difference between the averages of the observed protein Z scores and dividing by the standard deviation of all the differences for that particular comparison. A Z-ratio that was higher than 1.96 was inferred as significant. Unsupervised Hierarchical Clustering was used to investigate clusters of samples. Pathway analysis was performed by DAVID program [46] [47].

### EnSpire^®^ cellular label-free platform

MSTO-211H cells were seeded in specially designed 384-well plate with highly precise optical sensors able to measure changes in light refraction resulting from dynamic mass redistribution (DMR) within the cell's monolayer. Change in the light refraction was indicated by a shift in wavelength.

### Experimental animals and ethics statement

#### Xenograft transplantation

MSTO-211H cells were pre-treated with the artichoke extract (50 μg/ml) for 24 hrs. Suspensions of 2 × 10^6^ MSTO-211H cells × mouse (*n* = 6) were subcutaneously injected in PBS 1x/Matrigel (BD Biosciences San Jose, CA, USA) into 6-weeks-old female CD1 mice (Charles River, Milan). Tumor volume was calculated by using the formula: V 1/2 × length × width^2^ (by electronic caliper).

#### Animal studies

CD1 mice were subcutaneously transplanted with MSTO-211H (2 × 10^6^). At the evidence of tumor progression (when tumor volume reached 60 mm^3^) animals were randomly divided in five groups. Drinking water and a complete pellet diet (GLP 4RF21, Mucedola) were supplied ad libitum. Three groups of mice (*n* = 6) were given the artichoke extract in drinking water at the following concentrations: 25, 50 and 75 mg/ml, whereas mice of the control group (*n* = 6) were given tap. In the last group, pemetrexed was injected intraperitoneally at the dose of 100 mg/kg for five consecutive days. Body weight and clinical signs of the mice were checked every 3 days. All tumorigenicity assays were performed according to the guidelines set by the internal ethical committee. At the end of the experiment tumor masses were collected and fixed in 10% buffered formalin.

#### Immunoistochemical analysis

Formalin-fixed and paraffin-embedded 5 μm sections from mice tumor sections were stained with haematoxylin and eosin or stained with anti-ki67 antibody (ab15580, Abcam). Seven fields chosen randomly from each sample were scored.

## SUPPLEMENTARY FIGURES AND TABLE


