# Supplementary material for: Cynara scolymus affects malignant pleural mesothelioma by promoting apoptosis and restraining invasion
Source: Oncotarget. 2015 Jun 22;6(20):18134–50. doi: 10.18632/oncotarget.4017 (PMC4627240; doi:10.18632/oncotarget.4017)
Supplement: Supplementary file 1 [file oncotarget-06-18134-s001.pdf]

SUPPLEMENTAY FIGURES AND TABLE

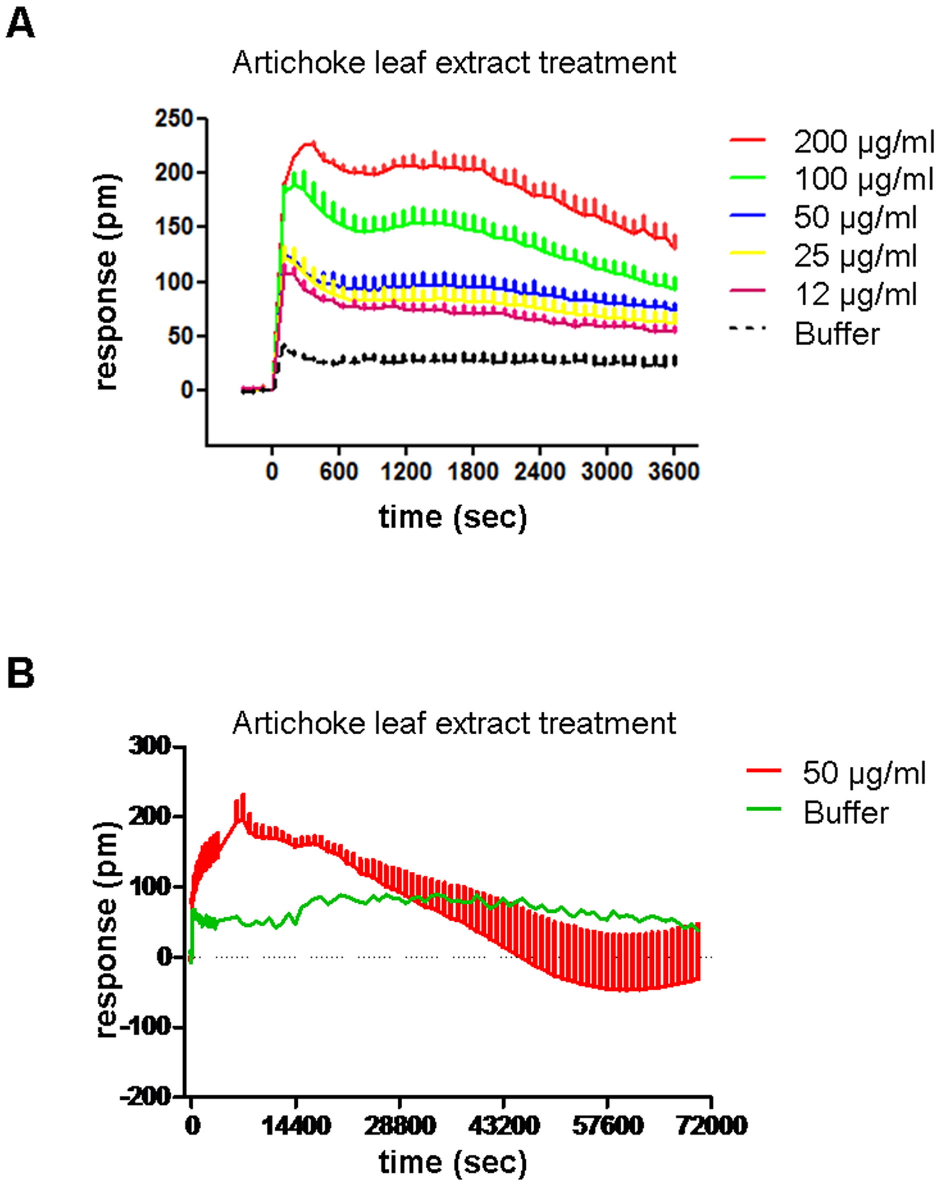

Supplementary Figure S1: Dose response of the artichoke leaf extract on MSTO-211H cells versus vehicle using label free EnSpire® analyzer technology. A. Short term response (0 – 3600 seconds) of MSTO-211H treated either with buffer or the artichoke leaf extract at the indicating doses. **B.** Long term response (0 – 72000 seconds) of MSTO-211H treated either with buffer or 50 µg/ml of the artichoke leaf extract.

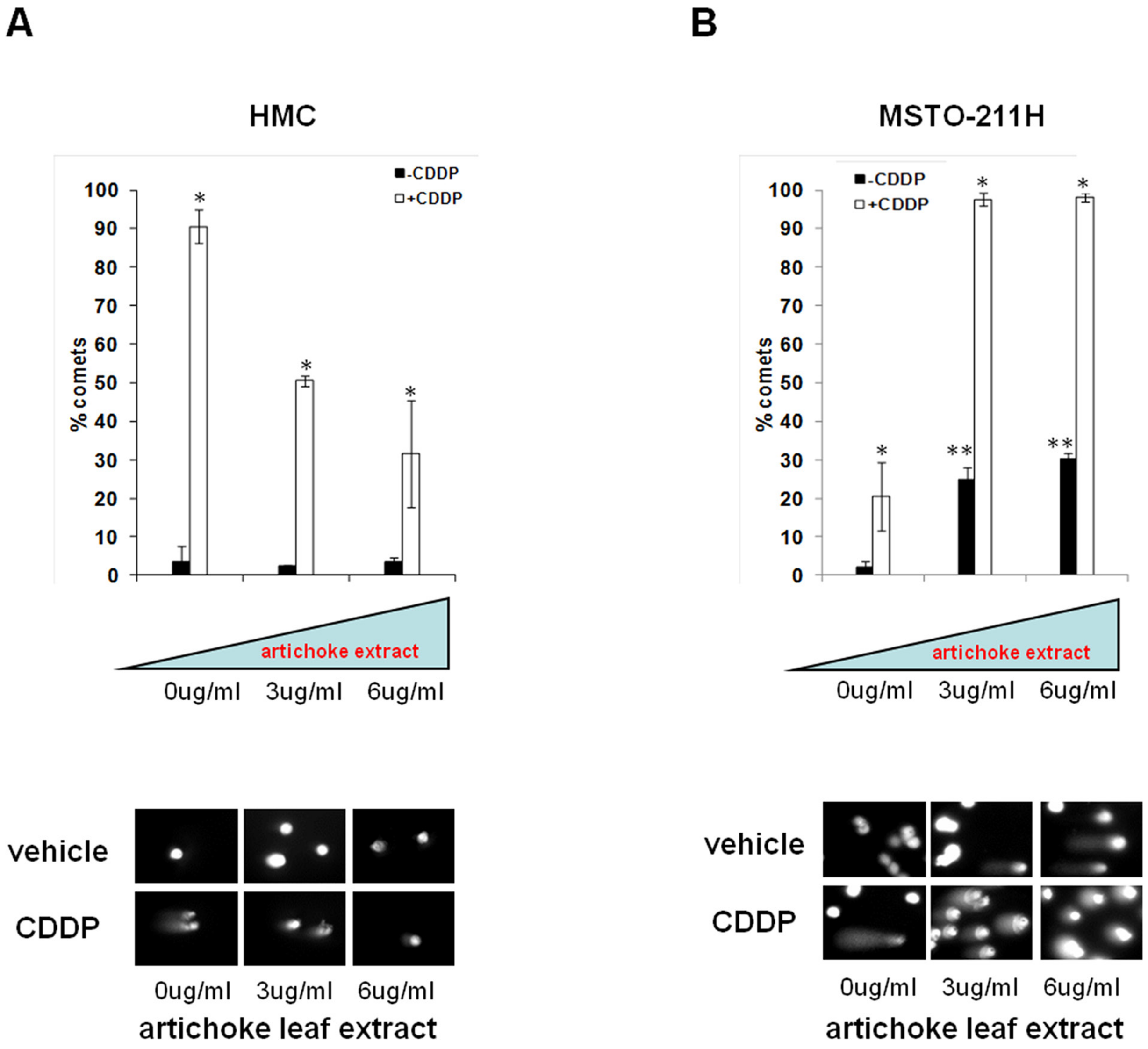

Supplementary Figure S2: The artichoke leaf extract-induced DNA damage of MPM cell lines. Upper part: histograms showing the percentage of comet obtained by treating HMC **A.** and MSTO-211H **B.** cell lines with the artichoke extract and with or without CDDP (7.5 µg/ml as indicated. Error bars represent mean +/– SD. Statistics (*t*-test): $p < 0.05$. Lower part: representative micrographs of cell comet from MSTO-211H and HMC cells treated as indicated.

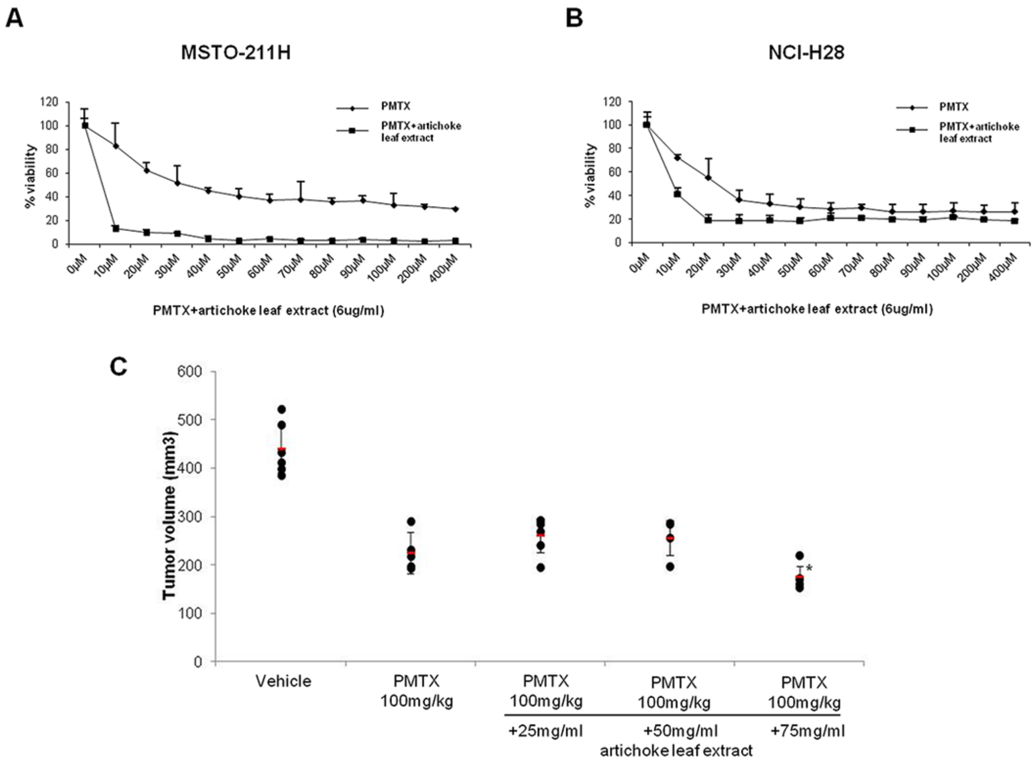

Supplementary Figure S3: The artichoke leaf extract sensitizes MPM cell lines to pemetrexed and potentiates its anti-tumoral effect. Viability of MSTO-211H **A.** and NCI-H28 **B.** cell lines treated for 72 hrs with either pemetrexed alone or in combination with 6 µg/ml of *Cynara scolymus* leaf extracts. Data are represented as mean +/- SD. Statistics (*t*-test): $p < 0.05$. **C.** The artichoke leaf extract beverage inhibits *in vivo* mesothelioma tumor progression. Tumour volumes of mice ($n = 6$) treated with either vehicle, pemetrexed or the artichoke extract in drinking water are reported. Statistics (*t*-test): $p < 0.05$.

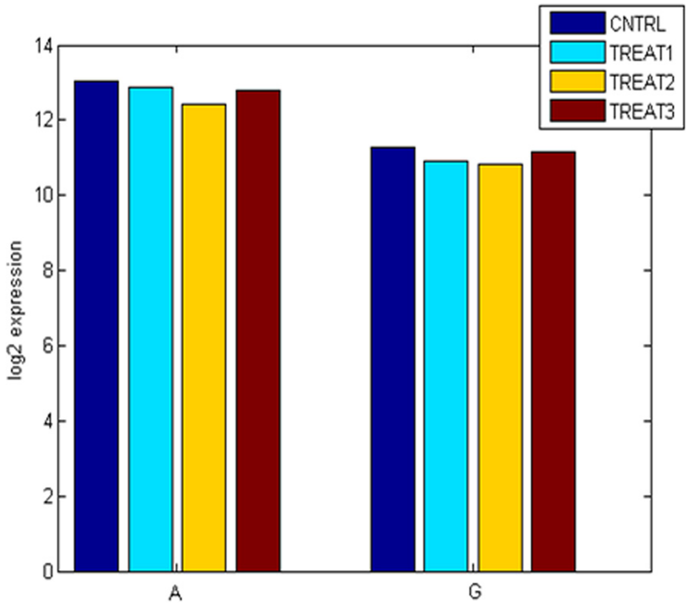

Supplementary Figure S4: Internal standard for phospho-array analysis. Levels of housekeeping proteins such as GAPDH and ACTIN.

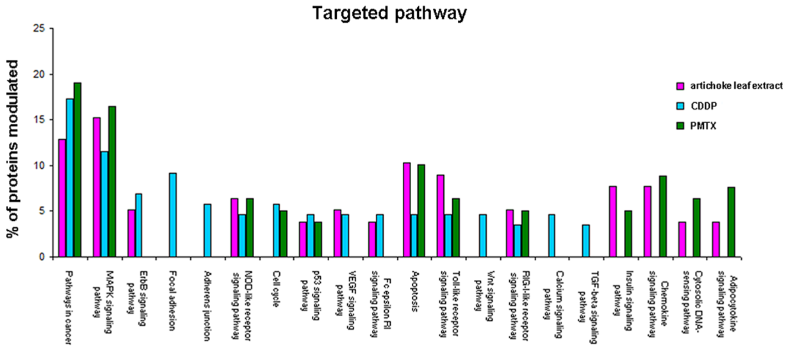

Supplementary Figure S5: Targeted prediction pathway analysis. The histograms represent the percentage number of proteins belonging to the difference pathways that result modulated after the three different drugs treatment compared to the vehicle. DAVID bioinformatic database was used for this analysis.

Supplementary Table S1: IC50s of the artichoke leaf extract treatment for different mesothelioma cell lines versus human mesothelial cells

IC50, artichoke leaf extract treatment	
MSTO-211H	21,02 μg/ml
MPP89	17,8 μg/ml
NCI-H28	28,08 μg/ml
HMC	33,3 μg/ml
